# Retention in Care Among People Living with HIV in Nigeria: A Systematic Review and Meta-analysis

**DOI:** 10.34172/jrhs.2024.153

**Published:** 2024-07-31

**Authors:** John O. Olawepo, Katherine O'Brien, Julia Papasodoro, Philip E. Coombs, Neha Singh, Shubhi Gupta, Aarushi Bhan, Babayemi O. Olakunde, Echezona E. Ezeanolue

**Affiliations:** ^1^Department of Health Sciences, Bouvé College of Health Sciences, Northeastern University, Boston, USA; ^2^Center for Translation and Implementation Research (CTAIR), University of Nigeria, Enugu, Nigeria; ^3^Department of Research and Instruction, Northeastern University Library, Northeastern University, Boston, USA; ^4^Department of Health Informatics, Khoury College of Computer Science, Bouvé College of Health Sciences, Northeastern University, Boston, USA; ^5^Department of Biology, College of Science, Northeastern University, Boston, MA, USA; ^6^Department of Population and Community Health, University of North Texas Health Science Center, Fort Worth, Texas, USA; ^7^Healthy Sunrise Foundation, Las Vegas, Nevada, USA

**Keywords:** Retention in care, Nigeria, Antiretroviral agents, Meta-analysis

## Abstract

**Background:** In 2021, Nigeria had an estimated 1.9 million people living with the human immunodeficiency virus (PLHIV) and 1.7 million (90%) on antiretroviral therapy (ART).

**Study Design:** A systematic review and meta-analysis.

**Methods:** This meta-analysis followed the Preferred Reporting Items for Systematic Reviews and Meta-analyses (PRISMA) 2020 guidelines. We searched PubMed, Embase, PsychINFO, CINAHL, Global Index Medicus, and Cochrane Library. Studies were included if they reported on ART retention in care among PLHIV in Nigeria. The random-effects meta-analyses were used to combine the studies that had complete retention data. The I^2^ statistic was used to assess the heterogeneity of the studies. A sensitivity analysis was then done by conducting a leave-one-out analysis. Afterward, data were analyzed using STATA version 18.

**Results:** The search yielded 966 unique articles, of which 52 studies met the inclusion criteria for the meta-analysis, and four experimental studies were split into their component arms. The total number of study participants was 563,410, and the pooled retention rate was 72% (95% CI: 67%, 76%; I^2^=99.9%; n=57). Sub-analysis showed that the Southeast region of Nigeria had the highest retention of 86% (95% CI: 78%, 92%), and the South-South had the lowest retention (58%; 95% CI: 38%, 79%).

**Conclusion:** In Nigeria, the pooled ART retention rate is less than optimal to achieve the UNAIDS goal of 95%, thus developing new models for ART retention is needed.

## Background

 Human immunodeficiency virus (HIV) is a complex disease that has led to significant morbidity and mortality since it was first identified in the 1980s.^1^ In 2021, 38.4 million adults and children globally were living with HIV, with 1.5 million of those individuals newly infected.^2^ Sub-Saharan Africa bears the highest burden with 51% of new HIV infections occurring in the region.^2^ Nigeria, Africa’s most populous country, had 1.9 million adults and children living with HIV in 2021, with an adult prevalence rate of 1.3%.^3^ Seventy-four thousand (74 000) new infections occurred in 2021, along with 51 000 deaths. Since 2010, new HIV infections in Nigeria have decreased by 39% which is less than the global average decrease.^3^

 Antiretroviral therapy (ART) is the mainstay of HIV treatment, and it prevents transmission by reducing the viral load of the infected individual to very low levels.^4^ Globally, around 85% of people living with HIV (PLHIV) are aware of their status, and 75% of PLHIV are adhering to ART.^2^ In Nigeria, 90% of PLHIV are on ART, and more adult women (97%) are receiving ART than adult men (94%) and children (31%). Moreover, 34% of pregnant women receive treatment for the prevention of mother-to-child transmission of HIV (PMTCT).^3^ Nigeria implements a “test and treat” policy, where all HIV-positive persons are eligible to receive ART, regardless of their CD4 + cell count. Although evidence suggests that most PLHIV will achieve viral load suppression within 6 months of ART initiation,^5,6^ it is unknown what proportion of PLHIV in Nigeria is retained on treatment long enough to attain and maintain viral suppression.

 Numerous studies have documented the importance of retention in HIV care for both viral suppression and mortality.^7-9^ Retention in care has contributed to averting mortality rates and has been linked to better health outcomes and safer sexual behaviors.^7,10^ However, there is no clear gold standard definition of retention, and the choice of retention metric is contextual. Retention is complex, challenging to define, and hard to measure.^11^ It is defined differently across various agencies. The Centers for Disease Control and Prevention (CDC) defines retention as two viral load tests or CD4 cell counts completed more than 3 months apart.^12^ In contrast, the World Health Organization (WHO) defines retention more loosely as the routine attendance of services such as clinic appointments and taking medication as per the patient’s needs.^13^ Likewise, the Health Resources Service Administration (HRSA) and Institute of Medicine (IOM) provide different definitions: two medical visits at least 90 days apart and at least two medical visits per 12 months, respectively.^12^

 Several studies have reported retention in care among PLHIV in Nigeria, but the differing definitions have created a knowledge gap about the true proportion of PLHIV retained on ART in Nigeria. Accordingly, this systematic review and meta-analysis strived to provide a pooled estimate of ART retention in Nigeria based on the literature published on this topic to better inform retention programs in the future.

## Methods

 This systematic review and meta-analysis followed the Preferred Reporting Items for Systematic Reviews and Meta-analyses (PRISMA) 2020 reporting guidelines.^14^ The review protocol was registered in PROSPERO on March 8, 2022.^15^ Searches were constructed by a librarian (PEC) with expertise in health sciences systematic reviews in September 2021. The following databases were subsequently searched: PubMed (Supplementary file 1, Table S1), Embase via Embase.com, PsycINFO (via EBSCO), CINAHL (via EBSCO), Global Index Medicus, and the Cochrane Library. Full search strings for each database are accessible at Northeastern University Library’s Digital Repository Service (https://repository.library.northeastern.edu/files/neu:0v838204v).^16^

###  Inclusion and exclusion criteria

 Eligibility criteria included studies with outcomes relating to retention in care for PLHIV who are on ART in Nigeria. Studies were excluded if they were not conducted in Nigeria, the population was not on ART, or there were no reported outcomes relating to retention in care. In addition, there were no restrictions on the publication date or study design, and eligible studies were limited to those published in the English language.

###  Data collection and extraction

 The systematic review screening software Rayyan^17^ was used to deduplicate results. A dual-blinded screening was conducted using Rayyan at both title/abstract (JP and KO) and full-text stages (KO and JP). Conflicts were resolved through team discussion and consensus (JOO, JP, and KO), and data extraction was conducted by four reviewers (JOO, JP, KO, and SG). The extracted information included first author, title, journal, year of publication, study design, study population, geopolitical region where the study was conducted, number of study participants (specifying arm, if randomized trial), age of participants (mean, median, and/or range), duration of study (year - year), follow-up time, number of retained participants, and retained proportion. Three reviewers (JP, KO, and SG) split up the articles equally and extracted data from each paper. The data were subsequently reviewed and organized (by JOO). In instances in which retention was measured multiple times during a study, we used the last (most recent) measurement.In addition, some of the studies did not state the number of people or proportion retained. Some of the studies provided figures for those lost to follow-up (LTFU), and these figures were extrapolated to compute the number retained on ART. For the age categorization, we aligned our analysis based on how the eligible studies categorized their participants, that is, participants ≤ 14 years were categorized as children, those aged 15-17 were adolescents, those aged 18-24 were young adults, and individuals aged 18-65 + were categorized as adults.

###  Quality review

 Included studies were assessed for risk of bias using the Effective Public Health Practice Project (EPHPP) quality assessment tool for quantitative studies.^18^ Articles were evaluated and rated strong, moderate, or weak based on selection bias, study design, confounders, blinding, data collection methods, and withdrawals and dropouts. A global rating was then assigned based on the criteria outlined in the EPHPP. Subsequently, articles received a global rating as good if there were no individual weak ratings in any category or moderate if there was only one weak rating in any individual category. The included articles were independently screened for quality assessment by two reviewers (JP and KO), and disagreements were resolved through discussion with JOO for the team to reach a consensus.

###  Statistical analysis

 The eligible articles were examined for summary statistical measures, primarily the proportion of PLHIV retained on ART. Only the studies that had the number retained (numerator) and the base population on ART (denominator) were included in the meta-analysis. Due to the widely varying nature of the studies, random effects meta-analyses were used to combine all the eligible studies. Experimental studies where participants had different exposure or intervention arms were split into their component arms for ease of the meta-analysis. Egger’s regression test was performed for asymmetry in the funnel plot for publication bias. The I^2^ statistic was used to assess the heterogeneity of the studies. A pooled effect size based on the individual effect sizes and their sampling variances was computed using the DerSimonian-Laird between-study variance estimators. We assessed influential cases/outliers to see if the pooled effect estimate we found was robust. A Baujat plot was used to detect studies that overly contributed to the heterogeneity in the meta-analysis. We also conducted a moderator analysis (Subgroup analysis). Sensitivity analysis was performed using a leave-one-out analysis. Sub-analyses were performed by age categories, geographical regions of Nigeria, participant follow-up time, and year of publication. Data were analyzed using STATA version 18.0.

## Results

###  Search results

**Figure 1:**
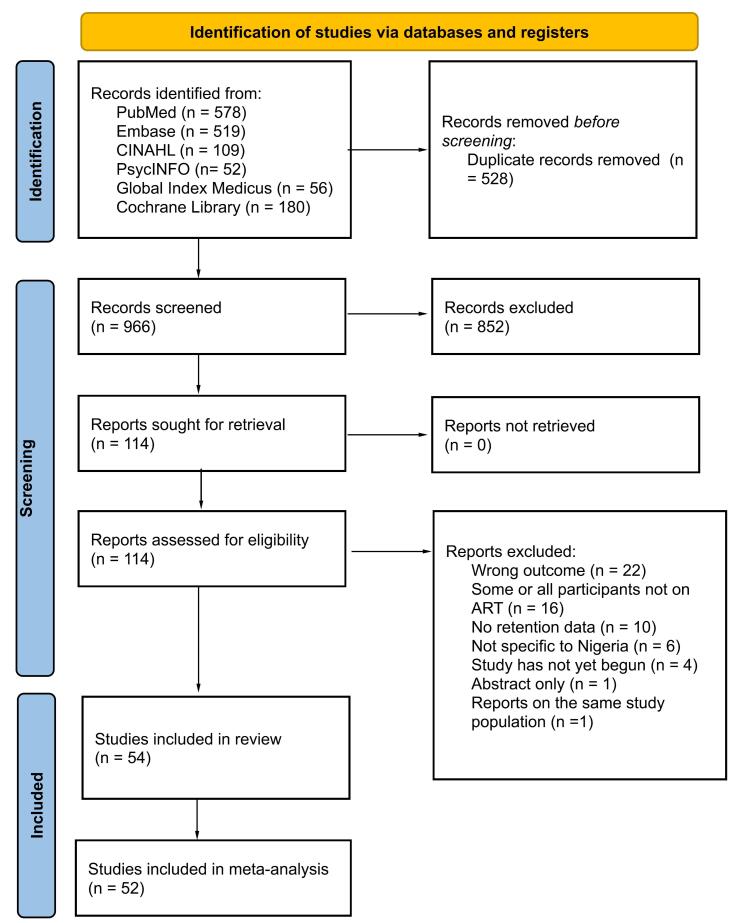
PRISMA flow diagram. *Note.* PRISMA: Preferred reporting items for systematic reviews and meta-analyses

 The initial search of the literature yielded 1,494 articles, of which 528 duplicate articles were removed through Rayyan.^17^ The remaining 966 articles were dually screened by abstract and titles, with 114 articles approved for full-text screening. Accordingly, 54 studies were included for the qualitative synthesis, and 52 for the meta-analytic synthesis (Supplementary file 1, Table S2)^19-72^ Sixty articles were excluded for the following reasons: wrong outcome (n = 22), some or all participants not on ART (n = 16), no retention data (n = 10), not specific to Nigeria (n = 6), study has not yet begun (n = 4), abstract only (n = 1), and reports on the same study population (n = 1) (see Figure 1 for the PRISMA Flow Diagram).

###  Description of included studies and qualitative synthesis

**Figure 2:**
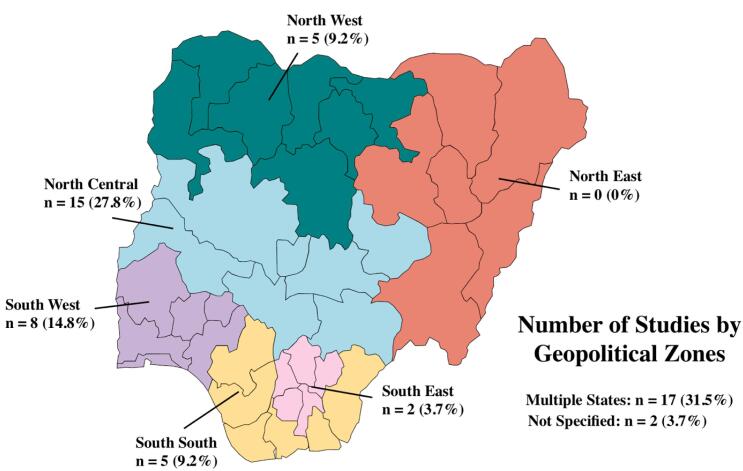
Number of studies by geopolitical zones

 The 54 eligible studies were published between 2005 and 2021. Seventeen studies were conducted across multiple states not enclosed within a specific geographical region in Nigeria, followed by 15 studies within states in the North-Central region. There were no identified studies conducted solely within states in the North-East region of Nigeria (Figure 2). Most of the studies were retrospective cohorts (n = 36, 67%), followed by prospective cohorts (n = 6, 11%), mixed designs (n = 5, 9%), quasi-experimental designs (n = 4, 7%), and randomized trials (n = 3, 6%). The total number of study participants from the 54 studies was 563 410. Furthermore, eight studies had more than 10 000 participants, but most of the studies (n = 20) had between 1000 and 10 000 participants. The follow up time of the studies ranged from 6 months to 14 years.

 Overall, we identified multiple definitions of retention among the eligible studies. For example, some authors defined retention as “being in care if the time between any two consecutive visits was ≤ 90 days and the time between the last visit and censor date was ≤ 180 days.”^23,24^ Others defined retention as “the number of people known to be alive and on ART 12 months after starting ART, including those who interrupted care (missed one or two appointments or drug pick-ups.)”^34^ Some authors further categorized their definition on how good or adequate the retention was. For example, “participants with a total of four visits (over 12 months) were categorized as having adequate retention while those who made less than four visits were categorized as having inadequate retention,”^38^ or “participants who visited the hospital and received ART refill at least once in each quarter for all the four quarters were classified as having good retention. Otherwise, they were classified as having poor retention.”^72^ Other authors also defined retention as “being alive and still on ART at the time of last appointment,”^42,63,68^ as “not having missed a scheduled appointment by more than 28 days,”^45^ as 2 HIV care-related visits to the clinic in each 24-week observation period,”^69^ or “having one or more clinic visits in the one year review period.”^71^

 Some contextual definitions were also identified. For example, in a differentiated care delivery model, retention was defined as “any stable ART patient who remained within their Community Anti-retroviral Groups (without default in routine clinic visits) at the end of the one-year follow-up period.”^41^ Additionally, in the eligible studies conducted in PMTCT settings, participants were defined as “fully retained-in-care at 6 months postpartum if the woman attended the 6-month postpartum visit (630 days) and did not miss any previous scheduled visit by more than 30 days (starting from ANC booking) or partially retained-in-care at 6 months postpartum if the woman attended the 6-month postpartum visit (630 days) but missed one or more earlier scheduled visits by more than 30 days.”^64^ Another study on PMTCT also defined maternal retention as “...by clinic attendance during the first 6-month postpartum. Participants with ≥ 3 of 6 expected monthly visits were considered retained.”^65,67^

###  Risk of bias and rating of study quality

 Using the EPHPP tool, the quality assessment showed that 40.7% (n = 22) of studies had moderate rating, 38.9% (n = 21) of studies had strong rating, and 20.4% (n = 11) of them had weak rating. Full results are available in Supplementary file 1 (Table S3).

###  Meta-analysis results

**Figure 3:**
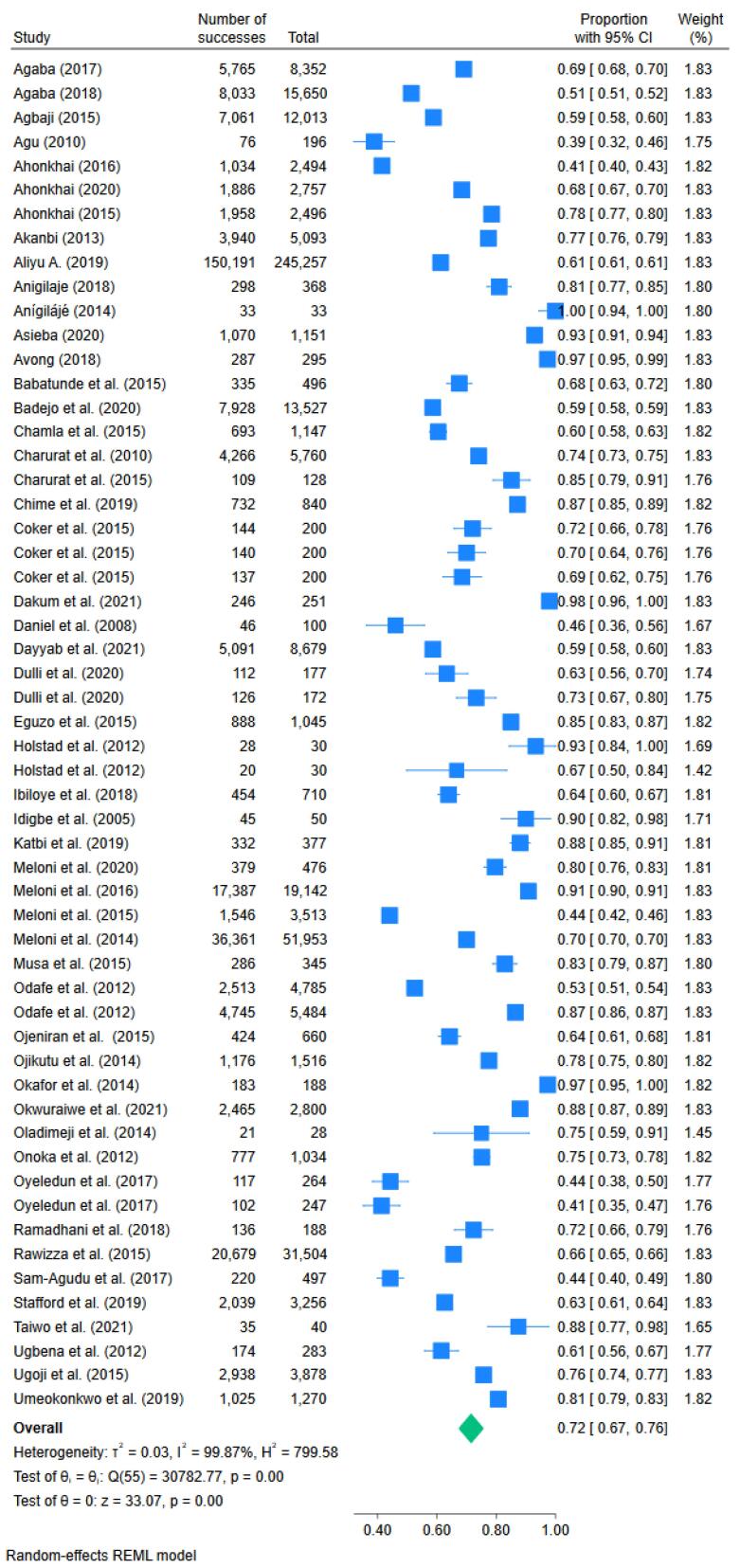
Forest plot for all included studies

**Figure 4:**
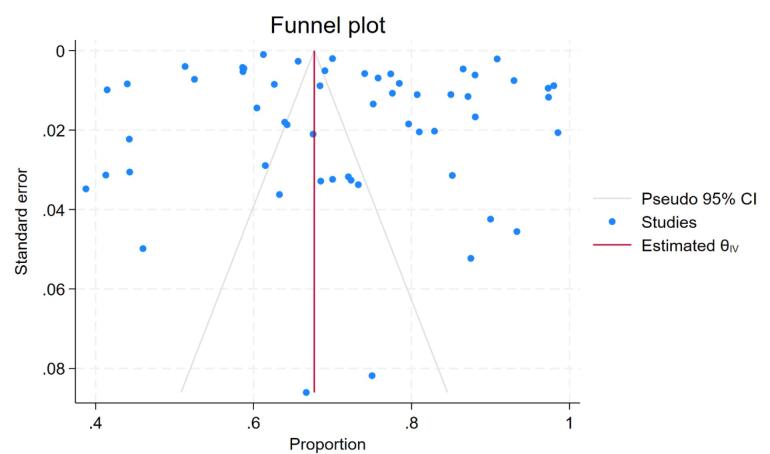
Funnel plot for included studies

**Figure 5:**
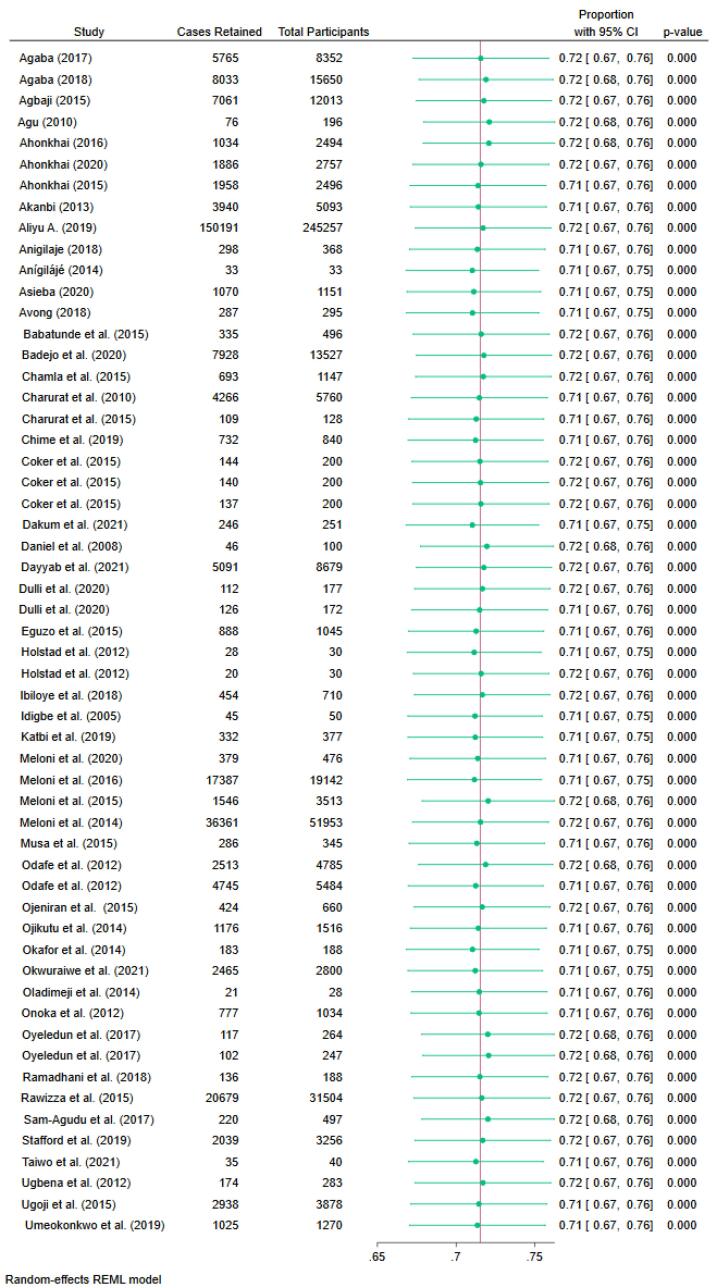
Sensitivity analysis results

 Fifty-two studies were included in the meta-analysis. Two out of the 54 studies were excluded from the meta-analysis because they only provided retention rates but no data on the number of patients retained.^27,42^ The pooled retention rate was 72% (95% confidence interval [CI]: 67,76; I^2^ = 99.9%; n = 57), as depicted in Figure 3, and retention ranged from 36.4% to 100%. There was no significant difference in proportion retained across publication years, age categories, region, or participant follow-up time. The Egger’s test (standard error = 1.27, z = 0.40, *P* = 0.6884) showed evidence of small-study effects (see funnel plot in Figure 4). Moreover, all the overall effect sizes from the leave-one-out analysis were close to the overall effect-size, and their confidence interval lines intersected with the overall effect size. This means that there are no studies that substantially influence the results of our meta-analysis (Figure 5).

###  Exploratory subgroup analysis

 Since heterogeneity between studies was 99.9%, we performed subgroup analysis by looking for between-study variance for such variables as age, year of publication, the geographic region in Nigeria, and participant follow-up time. Geographic region and participant follow-up time indicated a moderating effect. For age categorization, studies including adolescents and adults (n = 14) had a pooled retention rate of 71%, while those including adults only (n = 32) had a pooled retention rate of 72%. There were no eligible studies with only adolescents or children. Concerning the geographical regions, studies from the Southeast region (n = 5) had the highest pooled retention rate of 86%, while studies from the South-South region (n = 3) had a pooled retention rate of 58%. For participant follow-up time, the highest pooled retention rate was among studies with a follow-up time of 6-12 months (75%; n = 28), while the lowest pooled retention rate was among studies with a follow-up time of > 24-48 months (65%; n = 3). For the year of publication, studies published between 2011 and 2015 (n = 26) had the highest pooled retention at 73%. A summary of these retention rates and sub-analyses can be found in Table 1.

**Table 1:** Exploratory Subgroup Analyses Results

**Category**	**Studies**	**Effect size (95% CI)**	**I**^2^	***P*** ** value**
Age group				
Adolescents and adults	14	0.71 (0.63, 0.80)	99.8%	< 0.001
Adolescents, children, and young adults	8	0.70 (0.59, 0.82)	99.6%	< 0.001
Adults only	32	0.72 (0.66, 0.78)	99.8%	< 0.001
All age groups	3	0.72 (0.57, 0.87)	99.9%	< 0.001
Geographical region				
North-Central	15	0.74 (0.65, 0.83)	99.8%	< 0.001
North-West	7	0.67 (0.57, 0.78)	99.3%	< 0.001
South-East	5	0.86 (0.78, 0.92)	98.0%	< 0.001
South-South	3	0.58 (0.38, 0.79)	96.1%	< 0.001
South-West	9	0.75 (0.64, 0.85)	98.7%	< 0.001
Multiple states/regions	18	0.67 (0.60, 0.75)	99.9%	< 0.001
Participant follow-up time (month)				
6-12	28	0.75 (0.69, 0.81)	99.3%	< 0.001
> 12-24	13	0.66 (0.57, 0.75)	99.9%	< 0.001
> 24-48	3	0.65 (0.39, 0.90)	100%	< 0.001
> 48	12	0.70 (0.62, 0.78)	99.8%	< 0.001
Year of publication				
2005-2010	4	0.62 (0.39, 0.86)	98.5%	< 0.001
2011-2015	26	0.73 (0.68, 0.79)	99.7%	< 0.001
2016-2021	27	0.71 (0.64, 0.78)	99.9%	< 0.001

## Discussion

 The main finding from the current study is that from the eligible studies published between 2005-2021, the pooled retention rate for PLHIV on ART in Nigeria is 72%, which is less than optimal to achieve the UNAIDS 95-95-95 goals.^73^ Simply starting treatment does not guarantee viral suppression, and retention in care is a critical bridge between the second 95 and the third 95 of the UNAIDS goals.^74,75^ When retention is less than optimal, the benefits of ART become less attainable at the population level, and patients are at a higher risk for treatment failure, drug resistance, and death. If a considerable proportion of PLHIV on ART are not actively and continuously engaged in treatment, new HIV infections will continue to occur, further hampering the move towards zero HIV transmission.^76^

 Our findings align with previous studies that estimated 77%, 75%, and 75% retention rates at 6, 12, and 36 months, respectively, among adult patients on ART in Nigeria.^77^ The retention rate in our study also compares with the estimated average retention rates for sub-Saharan Africa.^77,78^ Despite the benefits of ART, studies have demonstrated that retention in care among PLHIV in Nigeria is affected by many factors, including non-disclosure of status, lack of social support, cost, travel time to the health facility, and perceived stigma.^79-81^ Improving retention will require multi-level interventions such as differentiated service delivery, education and behavior reminders, and community/peer support interventions to address these barriers.^82-84^

 The current study also found differences in effect sizes when categorized by participant follow-up time, indicating that research protocols may influence retention data. Retention rates are higher within the first six months and decline over time.^77-78^ In our meta-analysis, the retention rate was highest in studies with 6-12 months of follow-up. Studies with > 48 months also recorded high retention rates, but no specific reason was identified for this higher rate.

 Retention was also lower in adolescents, children, and young adults. Compared with older PLHIV, adolescents, children, and young adults are more likely to experience factors such as stigma, discrimination, and financial barriers that limit retention in care. It is important to note that limited studies have evaluated retention interventions among adolescents, children, and young adults.^84-85^ Hence, effective interventions to address retention among these vulnerable groups are urgently needed. Among the eligible articles in this study, only two studies were conducted among adult men who had sex with men, and one was conducted among the elderly. There were no studies on female sex workers, and this is another gap that could be filled by future studies.

 Furthermore, among the eligible studies included in this meta-analysis, there were no studies focused solely on the states within the Northeast geographical region. Although some states in the region were included in multi-state studies, we were not able to disentangle the data for analysis. This points to a potential disparity in research among PLHIV in this region and should be a focus for further exploration. Regional differences in the retention rates observed in this study may reflect the health-seeking behavior and geographic access to HIV care across the regions.

 Additionally, multiple thematically different definitions were identified from the 54 studies, indicating a lack of agreement on the definition of retention in care. This challenge further highlights the fact that retention in HIV care is a complex issue. Although the challenge of multiple definitions is not a new problem,^86-88^ metrics for measuring retention have included visit consistency, missing visits, gaps in care, and visit adherence.^11,12,89,90^ The diverse definitions of retention lead to the inability to accurately compare different strategies for improving retention, thus complicating efforts to improve retention programs and subsequent viral suppression. Therefore, we recommend that a standard definition of retention in care should be widely implemented in HIV studies. This will be crucial for ensuring that future research on retention in care can be more easily compared, and thus new interventions for increasing retention could be developed, implemented, and rigorously evaluated.

 Before this study, there has been no synthesis of published data examining retention in care among PLHIV on ART in Nigeria. This review is the first to systematically review the literature and produce an estimate of the true retention in care of the population, which will be important for designing retention programs in Nigeria in the future. In addition, we have identified geopolitical zones where research is inadequate, as well as subgroups where retention is sub-optimal.

 This study is subject to some limitations. We could not clearly identify the number of people retained for two studies; hence, we documented the proportion as stated by the authors, but we did not include the two papers in the meta-analysis. Secondly, since we did some extrapolation of retention numbers from the LTFU number presented by some studies, some of the extrapolations may not have been 100% accurate. However, we are confident that these extrapolations are close estimates. Third, the varying definitions of retention made it extremely difficult to categorize the papers sufficiently. Finally, the search was conducted in English language only. Despite that there are many other languages spoken in Nigeria, nearly all academic work on HIV in Nigeria is published in English. It is very unlikely that a paper about HIV in the Nigerian population will be published in any language other than English.

**Highlights**

- Retention in care on antiretroviral therapy (ART) in Nigeria from 2005 to 2021 is 72%.
- Retention was highest in the Southeast region with 86% retention.
- Retention was highest between 6-12 months on ART.
- Retention improved from 2005-2010 when it was 62% to 2016-2021 when it was 71%.

## Conclusion

 Although there has been progress in HIV case identification and enrollment in treatment, optimal retention while on ART is key to achieving viral suppression and ending the HIV epidemic. Our findings lead us to conclude that retention in care while on ART in Nigeria is 72%, with age and regional variations. This calls for targeted interventions to increase retention in care among PLHIV. Moreover, to further the research agenda on retention in care, HIV practitioners and researchers should agree on a universal definition of retention in care to ensure the standardization of study results.

## Acknowledgements

 The authors would like to thank the Institute for Health Equity and Social Justice Research, Northeastern University, Boston, USA.

## Authors’ Contribution

**Conceptualization:** John O. Olawepo, Echezona E. Ezeanolue.

**Data curation:** Philip E. Coombs, Katherine O’Brien, Julia Papasodoro, Shubhi Gupta, John O. Olawepo.

**Formal analysis:** Neha Singh, John O. Olawepo, Katherine O’Brien, Julia Papasodoro.

**Funding acquisition:** Katherine O’Brien, Julia Papasodoro, John O. Olawepo.

**Investigation:** John O. Olawepo, Katherine O’Brien, Julia Papasodoro, Shubhi Gupta, Aarushi Bhan.

**Methodology:** John O. Olawepo, Philip E. Coombs, Babayemi O. Olakunde, Echezona E. Ezeanolue.

**Project administration:** John O. Olawepo, Katherine O’Brien, Julia Papasodoro.

**Resources:** John O. Olawepo, Katherine O’Brien, Julia Papasodoro.

**Software:** Philip E. Coombs.

**Supervision:** John O. Olawepo.

**Validation:** John O. Olawepo, Neha Singh, Philip E. Coombs.

**Visualization:** Katherine O’Brien, Julia Papasodoro, Neha Singh.

**Writing–original draft:** Katherine O’Brien, Julia Papasodoro, John O. Olawepo.

**Writing–review & editing:** John O. Olawepo, Katherine O’Brien, Julia Papasodoro, Philip E. Coombs, Neha Singh, Shubhi Gupta, Aarushi Bhan, Babayemi O. Olakunde, Echezona E. Ezeanolue.

## Competing Interests

 The authors have no conflicts of interest to declare.

## Ethical Approval

 Ethical approval was not needed for this study since it is a systematic review and meta-analysis.

## Funding

 Katherine O’Brien and Julia Papasodoro received undergraduate student funding from the PEAK Ascent Award from Northeastern University, Boston, USA.

## Supplementary Files

Supplementary file 1 contains Tables S1-S3.
