# Supplementary material for: Retention in Care Among People Living with HIV in Nigeria: A Systematic Review and Meta-analysis
Source: J Res Health Sci. 2024 Jul 31;24(3):e00618. doi: 10.34172/jrhs.2024.153 (PMC11380731; doi:10.34172/jrhs.2024.153)
Supplement: Supplementary file 1 — contains Tables S1-S3. [file jrhs-24-e00618-s001.pdf]

doi:10.34172/jrhs.2024.153
JRHS 2024; 24(3):e00618

Supplementary Table 1: Sample search terms

Database	Search String
Pubmed (578 results)	((("HIV Infections" [Mesh] OR "HIV" [All Fields] OR “human immunodeficiency virus” [All Fields] OR "PLHIV" [All Fields]) AND (“Retention in Care” [Mesh] OR “Treatment Adherence and Compliance” [Mesh] OR “Lost to Follow-Up” [Mesh] OR “Retention” [All Fields] OR “Retained” [All Fields] OR “Loss to follow up”[All Fields] OR “LTFU”[All Fields] OR “lost to follow up” OR “Drop off from care”[All Fields] OR “HIV care continuum”[All Fields] OR “adherence”[All Fields] OR “attrition”[All Fields] OR “compliance” [All Fields])) OR ((“Retention in Care” [Mesh] OR “Treatment Adherence and Compliance” [Mesh] OR “Lost to Follow-Up” [Mesh] OR “Retention”[All Fields] OR “Retained”[All Fields] OR “Loss to follow up”[All Fields] OR “LTFU”[All Fields] OR “lost to follow up” [All Fields] OR “Drop off from care”[All Fields] OR “HIV care continuum”[All Fields] OR “adherence”[All Fields] OR “attrition”[All Fields] OR “compliance” [All Fields]) AND ("Antiretroviral Therapy, Highly Active" [Mesh] OR “Anti-Retroviral Agents” [Mesh] OR "HIV Infections/drug therapy" [Mesh] OR “ART”[All Fields] OR “HAART” [All Fields] OR “antiretroviral therapy”[All Fields] OR “antiretroviral treatment”[All Fields] OR “ARV” [All Fields] OR “ARVs” [All Fields] OR “HIV drug therapy”[All Fields] OR “HIV drugs” [All Fields] OR “HIV treatment”[All Fields]))) AND (“Nigeria” [Mesh] OR “Nigeria” [All Fields] OR “Nigerian” [All Fields] OR “Nigerians” [All Fields])

Supplementary Table 2: Study Characteristics of included studies

S/N	Author, Year	Study design	Geographic Region in Nigeria	Age Categories	Median age in years [IQR] - Except otherwise stated	Duration of study (Year - Year)	Participant Follow-up Time (Years)	Number retained (n/N)	Proportion retained (%)
1	(Agaba et al., 2017)[19]	Retrospective Cohort	North Central	Adolescents and Adults	35 [±8] (Mean)	2004-2012	2	5765/8352	69%
2	(Agaba et al., 2018)[20]	Retrospective Cohort	North Central	Adult	33 (28, 40)	2008-2012	3	8033/15,650	46.4%
3	(Agbaji et al., 2015)[21]	Retrospective Cohort	North Central	Adult	35 (30, 42)	2004-2012	8.5	7061/12,013	58.7%
4	(Agu et al., 2010)[22]	Retrospective Cohort	South-South	Adult	33.6 (Mean)	2005-2007	1.5	19/49	38.8%
5	(Ahonkhai et al., 2015)[23]	Retrospective Cohort	North-West	Adolescents and Adult	32 (27, 39)	2009-2011	1.9	40/51	78%
6	(Ahonkhai et al., 2016)[24]	Retrospective Cohort	North-West	Adolescents and Adult	Not stated	2009-2011	1.8	1034/2494	41.5%
7	(Ahonkhai et al., 2020)[25]	Retrospective Cohort	South-West	Adults	36 [pre user fees]; 37 years [Post user fees] (Mean)	2012-2015	2	1886/2757	68.4%
8	(Akanbi et al., 2013)[26]	Retrospective Cohort	North Central	Adults	35.1 [± 9.1] (Mean)	2006-2011	Not clear	3940/5093	77.4%
9	(Aliyu et al., 2019)[27]	Longitudinal Analysis / Quality of Care Audit	Multiple Regions	Not Specified	Not Clear	2008-2012	1	Not Stated	97%

10	(Aliyu et al., 2019)[28]	Retrospective Cross-sectional	Multiple Regions	Children, Adolescents, and Adult	1 - 65+ (Range)	2004-2017	13	150,191 / 245,257	61.2%
11	(Anígilájé et al., 2014)[29]	Mixed Method	North Central	Children	5 (3.5, 8.0)	2011-2012	1	33/33	100%
12	(Anigilage et al., 2018)[30]	Retrospective Cohort	North Central	Children	5.63 (3, 8)	2010-2013	1	298 / 368	80.9%
13	(Asieba et al., 2021)[31]	Retrospective data analysis	Multiple Regions	Adults	38.9 [± 9.7] (Mean)	2016-2018	1	1070/1151	93%
14	(Avong et al., 2018)[32]	Prospective Cohort	North Central	Adult	35 (30, 41)	2016-2017	1	287/295	97.3%
15	(Babatunde et al., 2015)[33]	Retrospective data analysis	South-West	Children, Adolescents, and Adults	36.3 [± 13.1] (Mean)	2005-2012	7	335/496	67.5%
16	(Badejo et al., 2020)[34]	Retrospective data analysis	North Central	Children, Adolescents, and Young Adults	21 (19, 23)	2015-2017	1	7928/13,527	58.6%
17	(Chamla et al., 2015)[35]	Retrospective data analysis	Multiple Regions	Children	3.5 (1.6, 7.2)	2011-2012	2	693/1142	60.7%
18	(Charurat et al., 2010)[36]	Retrospective data analysis	Multiple Regions	Adult	35 (29, 41)	2005-2006	1.5	4266/5760	74%
19	(Charurat et al., 2015)[37]	Prospective Cohort	North Central	Adolescents and Adults (Male only, MSM)	Not Clear	2013 - 2014	1	109/128	85%
20	(Chime et al., 2019)[38]	Cross-sectional and retrospective data analysis	South-East	Adults	38.5 [± 9.8] (Mean)	2016	1	732/840	87.1%
21	(Coker et al., 2015)[39]	Randomized Control Trial	North-West	Adults	32	2006-2008	0.75	421/600	70%
23	(Dakum et al., 2020)[40]	Retrospective Cohort	Multiple Regions	Adult (Elderly)	57.1 (52, 60)	2004-2018	14	62,821/101,652	62%

22	(Dakum et al., 2021)[41]	Quasi-experimental (one group pre-post)	North Central	Adults	38 (± 9.5] (Mean)	2017-2018	1	246/251	98%
24	(Dalhatu et al., 2016)[42]	Retrospective Cohort	Not Clear	Adolescents and Adult	33 (28, 40)	2004-2012	>7	Not Clear	36.4%
25	(Daniel et al., 2008)[43]	Retrospective Cohort	South-West	Adolescents and Adults	35.7 [± 11.4] (Mean)	2000-2003	2	46/100	46%
26	(Dayyab et al., 2021)[44]	Retrospective Cohort	North-West	Adult	34 (Mean)	2005-2018	14	5091/8679	58.7%
27	(Dulli et al., 2020)[45]	Randomized Control Trial	South-South	Adolescents and Young Adults	21.3 [± 2.3] (Mean)	2019	0.83	238/324	74.0%
28	(Eguzo et al., 2015)[46]	Retrospective Cohort	South-East	Adolescents and Adult	37	2008-2013	5	888/1045	85%
29	(Holstad et al., 2012)[47]	Quasi-experimental (two group post-test only)	South-West	Adults (Women only)	30.7 [± 5.9] (Mean)	2008	0.5	48/60	80%
30	(Ibiloye et al., 2018)[48]	Retrospective Cohort	North Central	Adult	30 (24, 35)	2016-2017	1	454 / 710	63.9%
31	(Idigbe et al., 2005)[49]	Prospective Cohort	South-West	Adolescents and Adult	34.5 (30, 60)	2002 (start date)	1	45/50	90%
32	(Katbi et al., 2019)[50]	Non-control interventional study	North Central	Adults	Not Clear	2016	0.5	332/ 377	88.1%
33	(Meloni et al., 2014)[51]	Retrospective data analysis	Multiple Regions	Adult	35 (29, 41)	2004-2011	1.5	36,361 / 51,953	70%
34	(Meloni et al., 2015)[52]	Retrospective data analysis	Multiple Regions	Children	3.7 (1.7, 7.1)	2005-2011	6	1546/3513	44%
35	(Meloni et al., 2016)[53]	Retrospective data analysis	Multiple Regions	Adolescents and Adult	35 (29, 41)	2004-2012	3	17,387 / 19,142	91%
36	(Meloni et al., 2020)[54]	Retrospective data analysis	North Central	Children and Adolescents	10-19 (Range)	2006-2016	10	379/476	79.6%

37	(Musa et al., 2015)[55]	Retrospective Cohort	North-West	Adult	40	2004-2014	10	286 / 34	83%
38	(Odafe et al., 2012)[56]	Retrospective Cohort	Multiple Regions	Adolescents and Adult	34 (28, 41)	2007-2010	3	2513 / 3299	76.2%
39	(Odafe et al., 2012)[57]	Retrospective Cohort	Multiple Regions	Children, Adolescents, and Adult	33 (27, 40)	2007- 2010	2	4,745 / 5,484	86.5%
40	(Ojeniran et al., 2015)[58]	Retrospective Cohort	South-West	Children	3.4 (1.5, 6.4)	2005-2011	7	424/660	64%
41	(Ojikutu et al., 2014)[59]	Retrospective Cohort	Not Clear	Children and Adolescents	0-18 (Range)	2002-2011	10	1176 / 1516	77.6%
42	(Okafor et al., 2014)[60]	Retrospective Cohort	South-East	Adults (Women only)	28.8 [± 4.1] (Mean)	2009-2011	1.5	183/188	97.3%
43	(Okwuraiwe et al., 2021)[61]	Retrospective comparison study	South-West	Adult	35.5 (25, 49)	2004-2014	7	2465/2800	88%
44	(Oladimeji et al., 2014)[62]	Retrospective Cohort	Multiple Regions	Adult	34 (29, 42)	2010-2012	0.67	21/28	75%
45	(Onoka et al., 2012)[63]	Retrospective data analysis	South-East	Adults	37.1 [± 9.3] - Public Facilities; 32.0 [± 12.7] - Private Facilities (Mean)	2007-2008	1	777/1034	75.1%
46	(Oyeledun et al., 2017)[64]	Cluster Randomized Control	Multiple Regions	Adult (Women only)	27 (23, 30)	2014-2016	0.5 (postpartum)	219/511	43%
47	(Ramadhani et al., 2018)[65]	Prospective Cohort	Multiple Regions	Adult (Male only, MSM)	Not Clear	2013-2017	0.5	136 / 188	72.3%
48	(Rawizza et al., 2015)[66]	Retrospective data analysis	Not Clear	Adult (Women only)	30.2 (26.8, 33.7)	2004-2014	1.5 (postpartum)	20,679 / 31,504	65.7%

49	(Sam-Agudu et al., 2017)[67]	Prospective paired cohort study	North Central	Adults (Women only)	Not Clear	2014-2015	0.5	220 / 497	44.3%
50	(Stafford et al., 2019)[68]	Retrospective Cohort	Multiple Regions	Adolescents and Adult	33 (27, 40)	2015-2017	1	2039/3256	62.6%
51	(Taiwo et al., 2021)[69]	Single-arm (pre and post) trial	South-West	Adolescents and Young Adult	19	Not clear	1	35/40	87.5%
52	(Ugbena et al., 2012)[70]	Cross sectional survey with retrospective data collection	North Central	Adult	34 (28, 40)	2008	1.25	174/283	61.5%
53	(Ugoji et al., 2015)[71]	Retrospective data analysis	Multiple Regions	Adolescents and Adults	Not Clear	2005-2010	1	2,938 / 3878	75.8
54	(Umeokonkwo et al., 2019)[72]	Comparative cross-sectional study	South-East	Adults	40.1 [± 9.9] (Mean)	2015-2015	1	1025/1270	80.7%

Supplementary Table 3: Quality review of eligible papers

S/N	Author, Year	Selection Bias	Study Design	Confounders	Blinding	Data Collection Methods	Withdrawals and Dropouts	Global Rating
1	Agaba et al., 2017[19]	Strong	Moderate	Strong	N/A	Strong	Moderate	Strong
2	Agaba et al., 2018[20]	Strong	Moderate	Strong	N/A	Strong	Weak	Moderate
3	Agbaji et al., 2015[21]	Strong	Moderate	Strong	N/A	Strong	Weak	Moderate
4	Agu et al., 2010[22]	Strong	Moderate	Weak	N/A	Weak	Weak	Weak
5	Ahonkhai et al., 2015[23]	Strong	Moderate	Strong	N/A	Strong	Weak	Moderate
6	Ahonkhai et al., 2016[24]	Strong	Moderate	Strong	N/A	Good	Weak	Moderate
7	Ahonkhai et al., 2020[25]	Strong	Moderate	Strong	N/A	Strong	Weak	Moderate
8	Akanbi et al., 2013[26]	Strong	Moderate	Strong	N/A	Strong	Moderate	Strong
9	Aliyu et al., 2019[27]	Strong	Moderate	Weak	N/A	Strong	Weak	Weak
10	Aliyu et al., 2019[28]	Moderate	Moderate	Strong	N/A	Moderate	Moderate	Strong
11	Anígilájé et al., 2014[29]	Weak	Moderate	Weak	N/A	Moderate	Strong	Weak
12	Anigilaje et al., 2018[30]	Moderate	Moderate	Moderate	N/A	Strong	Strong	Strong
13	Asieba et al., 2021[31]	Strong	Moderate	Strong	N/A	Moderate	Strong	Strong
14	Avong et al., 2018[32]	Weak	Moderate	Weak	N/A	Strong	Strong	Weak
15	Babatunde et al., 2015[33]	Moderate	Moderate	Weak	N/A	Strong	Weak	Weak
16	Badejo et al., 2020[34]	Moderate	Moderate	Strong	N/A	Strong	Weak	Moderate
17	Chamla et al., 2015[35]	Strong	Moderate	Moderate	N/A	Strong	Moderate	Strong
18	Charurat et al., 2010[36]	Strong	Moderate	Strong	N/A	Strong	Moderate	Strong
19	Charurat et al., 2015[37]	Moderate	Moderate	Strong	N/A	Moderate	Moderate	Strong
20	Chime et al., 2019[38]	Strong	Moderate	Strong	N/A	Strong	Strong	Strong
21	Coker et al., 2015[39]	Strong	Strong	Strong	Weak	Strong	Moderate	Moderate
22	Dakum et al., 2020[40]	Strong	Moderate	Weak	N/A	Strong	Moderate	Moderate
23	Dakum et al., 2021[41]	Moderate	Moderate	Weak	N/A	Strong	Strong	Weak

24	Dalhatu et al., 2016[42]	Strong	Moderate	Strong	N/A	Strong	Weak	Moderate
25	Daniel et al., 2008[43]	Moderate	Moderate	Weak	N/A	Weak	Weak	Weak
26	Dayyab et al., 2021[44]	Strong	Moderate	Strong	N/A	Strong	Weak	Moderate
27	Dulli et al., 2020[45]	Strong	Strong	Strong	Weak	Strong	Moderate	Moderate
28	Eguzo et al., 2015[46]	Strong	Moderate	Moderate	N/A	Weak	Moderate	Moderate
29	Holstad et al., 2012[47]	Strong	Strong	Strong	Weak	Strong	Strong	Moderate
30	Ibiloye et al., 2018[48]	Moderate	Moderate	Strong	N/A	Moderate	Moderate	Strong
31	Idigbe et al., 2005[49]	Moderate	Moderate	Weak	N/A	Moderate	Strong	Moderate
32	Katbi et al., 2019[50]	Moderate	Moderate	Weak	N/A	Weak	Strong	Weak
33	Meloni et al., 2014[51]	Strong	Moderate	Strong	N/A	Strong	Moderate	Strong
34	Meloni et al., 2015[52]	Strong	Moderate	Strong	N/A	Strong	Weak	Moderate
35	Meloni et al., 2016[53]	Strong	Moderate	Strong	N/A	Strong	Moderate	Strong
36	Meloni et al., 2020[54]	Moderate	Moderate	Moderate	N/A	Strong	Moderate	Strong
37	Musa et al., 2015[55]	Strong	Moderate	Strong	N/A	Moderate	Strong	Strong
38	Odafe et al., 2012[56]	Strong	Moderate	Strong	N/A	Strong	Weak	Moderate
39	Odafe et al., 2012[57]	Moderate	Moderate	Strong	N/A	Strong	Strong	Strong
40	Ojeniran et al., 2015[58]	Moderate	Moderate	Strong	N/A	Strong	Weak	Moderate
41	Ojikutu et al., 2014[59]	Strong	Moderate	Strong	N/A	Strong	Moderate	Strong
42	Okafor et al., 2014[60]	Strong	Moderate	Weak	N/A	Weak	Strong	Weak
43	Okwuraiwe et al., 2021[61]	Weak	Moderate	Weak	N/A	Strong	Strong	Weak
44	Oladimeji et al., 2014[62]	Moderate	Moderate	Weak	N/A	Strong	Strong	Moderate
45	Onoka et al., 2012[63]	Moderate	Moderate	Strong	N/A	Moderate	Moderate	Strong
46	Oyeledun et al., 2017[64]	Strong	Strong	Strong	Weak	Strong	Weak	Weak
47	Ramadhani et al., 2018[65]	Moderate	Moderate	Strong	N/A	Moderate	Moderate	Strong
48	Rawizza et al., 2015[66]	Moderate	Moderate	Weak	N/A	Strong	Moderate	Moderate
49	Sam-Agudu et al., 2017[67]	Strong	Moderate	Strong	N/A	Strong	Weak	Moderate
50	Stafford et al., 2019[68]	Strong	Moderate	Strong	N/A	Strong	Moderate	Strong
51	Taiwo et al., 2021[69]	Strong	Moderate	Weak	N/A	Moderate	Strong	Moderate
52	Ugbena et al., 2012[70]	Moderate	Moderate	Weak	N/A	Moderate	Moderate	Moderate
53	Ugoji et al., 2015[71]	Strong	Moderate	Strong	N/A	Moderate	Moderate	Strong
54	Umeokonkwo et al., 2019[72]	Strong	Moderate	Strong	N/A	Moderate	Strong	Strong
